# Damage Detection in Glass Fibre Composites Using Cointegrated Hyperspectral Images

**DOI:** 10.3390/s24061980

**Published:** 2024-03-20

**Authors:** Jan Długosz, Phong B. Dao, Wiesław J. Staszewski, Tadeusz Uhl

**Affiliations:** Department of Robotics and Mechatronics, Faculty of Mechanical Engineering and Robotics, AGH University of Krakow, 30-059 Krakow, Poland; phongdao@agh.edu.pl (P.B.D.); w.j.staszewski@agh.edu.pl (W.J.S.)

**Keywords:** hyperspectral imaging, glass fibre-reinforced plastics, damage detection

## Abstract

Hyperspectral imaging (HSI) is a remote sensing technique that has been successfully applied for the task of damage detection in glass fibre-reinforced plastic (GFRP) materials. Similarly to other vision-based detection methods, one of the drawbacks of HSI is its susceptibility to the lighting conditions during the imaging, which is a serious issue for gathering hyperspectral data in real-life scenarios. In this study, a data conditioning procedure is proposed for improving the results of damage detection with various classifiers. The developed procedure is based on the concept of signal stationarity and cointegration analysis, and achieves its goal by performing the detection and removal of the non-stationary trends in hyperspectral images caused by imperfect lighting. To evaluate the effectiveness of the proposed method, two damage detection tests have been performed on a damaged GFRP specimen: one using the proposed method, and one using an established damage detection workflow, based on the works of other authors. Application of the proposed procedure in the processing of a hyperspectral image of a damaged GFRP specimen resulted in significantly improved accuracy, sensitivity, and F-score, independently of the type of classifier used.

## 1. Introduction

Glass fibre-reinforced polymers (GFRPs) are a type of composite consisting of glass fibres embedded in a polymer matrix. Using GFRPs in place of more traditional engineering materials, such as polymers, metals, or ceramics has become more and more widespread, due to a number of advantageous factors [1,2]. Among them, a few of the most important factors are their exceptional strength to weight ratio, high resistance to harsh environmental conditions, high durability, and ability to be manufactured into complex shapes using a range of processes. Due to these advantages, GFRPs are one of the most widely utilized composite materials across all the fields of engineering, e.g., the manufacturing of airship fuselage and wing parts, ship hulls, auto body parts, printed circuit boards, pressure tanks, concrete reinforcement bars, and blades of wind turbines. Despite their numerous advantages, successfully using GFRPs also poses a challenge, due to the increased number of failure modes of composite materials [3].

The most common and serious failure modes in GFRPs are the development of cracks and delaminations. Typical causes of the damage include manufacturing-induced defects, cyclic and dynamic loading, and exposure to environmental factors [4,5], which are the result of varying and difficult operating conditions. The presence of manufacturing-induced defects leads to failure in the early stages of their exploitation by significantly reducing the mechanical properties of the material. However, these kinds of defects can be avoided by employing an extensive post-manufacturing quality control. The loading and environmental issues are much harder to circumvent. Some of these problems can be avoided in the early stages of designing GFRP structures, especially by supporting the design with the data gathered from already deployed structures and provided by simulations, which are able to obtain strikingly accurate results by the employment of improved simulation techniques [6,7]. While this process is vital to preventing the damage, operational conditions of GFRP structures can be unpredictable. Because of this, the use of structural health monitoring (SHM) and damage detection techniques are employed to improve safety and power generation efficiency, and to minimize the maintenance cost.

Most of the damage detection techniques are based on strain measurement, acoustic emission, ultrasound, vibration, thermography, or vision [8]. Strain measurement-based damage detection utilizes strain sensors, either installed on the surface or integrated into the blade itself, and infer the presence of damage by detecting anomalies in the strain signal [9]. The drawback to this approach is that the accuracy of damage detection is dependent on the distance between the damage and the sensor. Acoustic emission events are detected by piezoelectric sensors installed on the blade of the turbine that register the elastic waves caused by the release of energy during the appearance of the damage [10]. The main challenges these systems face are distinguishing between the acoustic emission events caused by the damage and the noise, and the high internal damping of GFRPs. Ultrasound-based methods are proven to reliably detect the damage using the interaction between the damage and the ultrasonic waves [11]. However, the ultrasonic tests are limited by the accessibility of the tested object, which is especially unfavourable in offshore turbines. Vibration-based methods aim to detect changes in mechanical properties with the use of modal characteristics [12]. It has been observed that these kinds of methods fail to detect damage in its early stages. The thermography-based approach aims to detect damage using remotely registered thermal gradients on the surface of the wind turbine blade; however, similarly to the vibration-based approach, it is unable to detect damage in its early stages, and is prone to being heavily influenced by environmental factors such as temperature and humidity [13].

One of the most promising advances in SHM and damage detection was made in the field of vision-based methods [14]. Some of the techniques used in vision-based methods include stereoscopic vision, digital image correlation, computational intelligence-based algorithms and hyperspectral imaging (HSI) [15,16]. Overall, the quality of results obtained using vision-based methods is dependent on the quality of lighting [17], which is understandably hard to control in real-life applications due to the environmental influences. Hyperspectral imaging also suffers from that drawback. However, due to the increased amount of data gathered in HSI, in comparison to the other vision-based methods, the filtering of such influence yields better results.

The current methods developed for overcoming the lighting issue in vision-based methods, and especially in hyperspectral imaging, have been advanced classification methods, such as classifiers operating on spectral–spatial data [18,19,20], where instead of classifying each pixel alone, the data about the neighbouring pixels and location of the pixel in question are also included in the learning data [21], which lowers the method’s sensitivity to imperfect lighting. This method, however, performs best in scenarios where different endmembers (classes) present on the hyperspectral image are of a different chemical composition from one another, resulting in a high separation of those classes. In cases where the physical properties, such as the presence of cracks or delaminations, are the endmembers, the spectral–spatial approach fails to distinguish between a higher intensity pixel being an endmember or being a result of the uneven lighting.

To resolve this issue, we propose a technique which utilizes cointegration analysis as a data conditioning tool, and which aims to minimize the influence of imperfect lighting on the performance of HSI-based damage detectors. To the best of the authors’ knowledge, the cointegration analysis has never been investigated in the literature to develop a data conditioning process for classification of hyperspectral images used for damage detection in GFRPs, while its proven usefulness in trend removal applications makes it an appealing option to explore for HSI processing.

## 2. Materials and Methods

### 2.1. Damage Detection Using Hyperspectral Imaging

In hyperspectral imaging, a series of monochromatic images is registered over a wide range of the electromagnetic spectrum. While a traditional digital photograph contains three channels, each corresponding to one of red, green, or blue colours, hyperspectral images can contain hundreds of channels, each channel containing information about a single wavelength of electromagnetic waves. Most commonly, hyperspectral images cover the visible part of the spectrum, around 380 to 700 nm, and a part of the infrared spectrum, in our case near infrared (NiR) and short-wave infrared (SWiR) at 700 to 2500 nm. Hyperspectral images are stored in data structures called hypercubes. Their dimensions are x×y×λ, where *x* and *y* are spatial dimensions, and λ is a spectral dimension.

**Figure 1:**
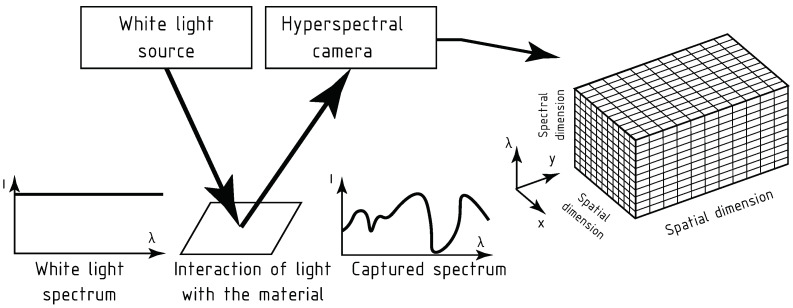
The process of hyperspectral imaging.

**Figure 2:**
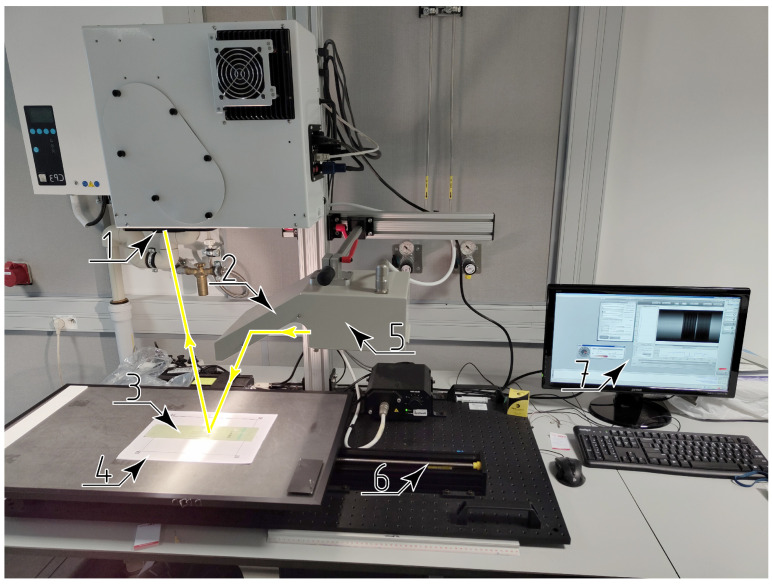
The hyperspectral camera setup with the trajectory of light in yellow: 1—The hyperspectral sensor, 2—the reflector, 3—the specimen, 4—the background with fiducial markers, 5—the illuminator, 6—the linear stage, 7—the preview monitor.

In the process of hyperspectral imaging, a specimen is lit with a white light by the illuminator. After the interaction of electromagnetic radiation with the material, the reflected light is registered with a hyperspectral camera and saved into a hypercube, as shown by a schematic in Figure 1, and in the photograph of the hyperspectral camera in Figure 2.

Hyperspectral imaging is based on the interaction of light with matter, primarily through the mechanisms of absorption and reflection. Absorption is mainly influenced by the chemical composition of the matter. Every element has a characteristic spectral signature that describes the intensity at which every wavelength in a given spectrum is absorbed. Thanks to this phenomenon, hyperspectral images contain chemical information about the scanned object, similar to the information that could be obtained using a spectrometric test. On the other hand, the phenomenon of reflection is primarily influenced by the physical features of the scanned object. Instead of applying to a select few of the wavelengths as is the case in chemical difference, the physical difference in the scanned object affects the whole spectrum with nearly the same intensity at every wavelength of the hyperspectral image.

Thanks to its ability to detect chemical and physical changes, HSI has found uses in the fields of medicine, agriculture, remote sensing, and many more [22,23]. In our study, we are focusing on the application of hyperspectral imaging in damage detection systems for GFRP composites.

The great amount of information and high dimensionality of hyperspectral images limits the use of conventional digital image processing techniques. One of the ways of circumventing this issue is the deployment of classification algorithms, either conventional, such as Adaptive Cosine Estimators (ACEs), or machine learning-based classification algorithms, such as support vector machines (SVMs) or artificial neural networks (ANNs).

While it has been proven that such systems can perform the task of damage detection well in laboratory conditions, in real-life applications, the issue of imperfect lighting becomes crucial. The change in the radiant flux of lighting results in the shift of the spectrum in a way that is similar to the presence of a physical feature, such as formation of a crack or delamination, which leads to an increase in false positive damage detections.

### 2.2. Cointegration Analysis

Hyperspectral images can be considered as multidimensional, stochastic, discrete signals. One of the properties that apply to such signals is its stationarity. A signal is considered to be strictly stationary when the probability density function (PDF) of a stochastic variable is constant over the signal’s duration. This definition often finds a limited use in signal processing, especially in the case of processes realized in real-world full of noise and non-ideal systems. For this purpose, a range of weaker criteria are utilized: the *N*-th order stationarity, and weak-, or wide-sense, stationarity. The process $X_{t}$ is classified as *n*-th degree stationary if it satisfies Equation (1):

(1) $$F_{X}\left(x_{t_{1+\tau }},\;...,\;x_{t_{n+\tau }}\right)=F_{X}\left(x_{t_{1}},\;...,\;x_{t_{n}}\right)\;\forall \;\tau ,t_{1},\;...,\;t_{n}\in R\;\wedge \forall \;n\in \left\{1,\;...,\;N\right\}$$

essentially limiting the global requirement to a requirement for *n* up to an order *N*.

The signal of weak-sense stationarity is only required to have a constant mean and autocovariance over its duration.

Other types of stationarity are the trend-stationary signals and difference-stationary signals. The signal is called trend-stationary if, after the removal of the deterministic trend, it becomes stationary [24]. The signal is called difference-stationary if it becomes stationary after one or few differentiations. Another more precise way of describing such a property is to state the order of integration of the signal. A signal of *d*-th order of integration is typically denoted as I(d). The order of integration can be thought of as the number of times the differentiation is required to be applied to transform the signal into a stationary signal. It is also a way of describing the presence of a unit root.

To confirm or deny the presence of a unit root, a range of statistical tests have been developed. Two of the most widely used unit root tests are the Dickey–Fuller test (AD test) and the Augmented Dickey–Fuller test (ADF test) [25].

In the AD test, a data series is represented as a 1st order autoregressive signal (AR(1)), which is represented as:

(2) $$X_{t}=\alpha +\rho X_{t-1}+\varepsilon_{t}$$

where $X_{t}$ is a signal at time *t*, α is a trend term, ρ is a parameter of the lagged term, and $\varepsilon_{t}$ is the error of the AR model. The value of α=0 signifies that the process is a random walk, there are no stochastic trends in a signal, and the value of α≠0 means that the process is a random drift.

The AD test is used to determine if the signal is integrated of order I(0) or I(1) [26], and decides between two hypotheses:

(3) $$\begin{array}{cc} H_{0}: & \;\rho =1\\ H_{1}: & \;\rho <1 \end{array}$$

The rejection of $H_{0}$ means that the signal is stationary in the wide sense—integrated of order I(1). The hypothesis can be rejected or confirmed after performing a *t*-test over the duration of the signal.

In practice, however, such an evaluation is improper, as both $X_{t}$ and $X_{t-1}$ could be non-stationary, thus transforming the t-distribution into a normal distribution, due to the normal central limit theorem. To bypass that issue, the AR model is constructed for the first differences of the signal, such that:

(4) $$X_{t}-X_{t-1}=\alpha +\left(\rho -1\right)X_{t-1}+\varepsilon_{t}$$

which can also be represented as:

(5) $$\Delta X_{t}=\alpha +\delta X_{t-1}+\varepsilon_{t}$$

Because $\Delta X_{t}$ is in essence a first difference of $X_{t}$, it is integrated of order I(0). If the $H_{0}$ holds true, then in the differenced model, a parameter δ=0, removing the potentially non-stationary term $X_{t-1}$ from the right-hand side of (5), thus allowing for calculation of the t-statistic. The calculated t-statistic is then evaluated against the critical value of the Dickey–Fuller distribution for a chosen significance level, which is the criterion for rejecting or approving the $H_{0}$.

The ADF test expands the AD test by allowing the use of higher order AR models [26]. This is achieved by using an additional lagged term in the AR model. For the ADF test, the model with one lagged term (AR(2)) takes the form of:

(6) $$\Delta X_{t}=\alpha +\delta_{0}X_{t-1}+\delta_{1}\Delta X_{t-1}+\varepsilon_{t}$$

or in general, for *n* lagged terms in AR(n+1) model:

(7) $$\Delta X_{t}=\alpha +\delta_{0}X_{t-1}+\sum_{i=1}^{n}\delta_{i}\Delta X_{t-i}+\varepsilon_{t}$$

The hypotheses for ADF are formulated as:

(8) $$\begin{array}{cc} H_{0}: & \;\delta_{0}=0\\ H_{1}: & \;\delta_{0}<0 \end{array}$$

Similarly to AD, rejection of $H_{0}$, on the grounds of the $\delta_{0}$ t-statistic absolute value being smaller than the critical value of the DF distribution’s critical value for a given significance level, indicates that the *X* series’ order of integration is less than I(1).

Cointegration analysis is based on a concept of stationarity [27]. The cointegrating relationship between two data series exists if

(9) $$X_{c}=\beta^{\prime }X$$

is integrated of order I(d−1), where β is the cointegrating vector, and X is a collection of $\left(X_{1},X_{2},\ldots ,X_{k}\right)$ time series integrated of order I(d). In essence, a cointegrating relationship exists if a linear combination of two or more non-stationary data series is itself stationary. In this context, cointegration analysis is used as a detrending tool to remove the influence of the environment [28,29].

After ensuring that all data series considered are integrated of the same order, and that the order is at least I(1), the next step is to test for the existence of cointegration relationships among them. For this purpose, a Johansen test is utilized [30].

The Johansen test for cointegration is a two-part procedure. In the first step, the signals are modelled using the Vector Error Correction Models (VECMs) [31]. There are a few variants of VECMs, and the difference between them are the presence or absence of terms representing the trend, and the constant, and the number of cointegrating vectors.

The process of estimating the VECM itself consists of four steps. The first step is to estimate a Vector Autoregressive Model (VAR) for the collection of signals $X_{t}$. The VAR model of rank *p* takes the form of [32]:

(10) $$X_{t}=\Phi D_{t}+\Pi_{1}X_{t-1}+\ldots +\Pi_{p}X_{t-p}+\varepsilon_{t}$$

where $D_{t}$ are the deterministic terms that consist of constant ($u_{0}$) and trend ($u_{1}t$) terms, and can be expanded as:

(11) $$D_{t}=u_{0}+u_{1}t$$

where Φ is the parameter of deterministic terms, and $\Pi_{i}$ are the parameters of lagged terms.

The VAR model is then transformed into a VECM model. VECMs are the extension of Error Correction Models for multivariate area, and are formulated as:

(12) $$\Delta X_{t}=\Phi D_{t}+\Pi X_{t-1}+\Gamma_{1}\Delta X_{t-1}+\ldots +\Gamma_{p-1}\Delta X_{t-p+1}+\varepsilon_{t}$$

where $D_{t}$ are the deterministic terms, Φ is the parameter of deterministic terms, and is constructed as $\Pi =\Pi_{1}+\ldots +\Pi_{p}-I_{n}$, Π is the matrix of long run impact, and Γ is the matrix of short-term impact, taking the form of $\Gamma_{k}=-\sum_{j=k+1}^{p}\Pi_{j}$ for k=1,…,p.

By transforming a VAR model into a VECM model, it is ensured that the variables integrated of order I(1) are encapsulated in the $\Pi X_{t-1}$ term; therefore, if the cointegrating relationship exists, it will be included in this term.

The rank of matrix Π:

(13) r=rank(Π)

represents the number of potential cointegrating vectors found. It can take a value between 0 and *n*.

The matrix Π is then factorized into two matrices:

(14) $$\Pi =AB^{T}$$

where *A* and *B* are (n×r) matrices of rank *r*. Matrix *B* is a collection of potential cointegrating vectors.

The second part of the Johansen test is to test found potential cointegrating vectors, using either the trace or maximum eigenvalue statistic. This test consists of m∈[0,r] stages, and in every stage, it challenges the null hypothesis that the number of cointegrating vectors $H_{0}:r=m$ with the alternative being $H_{1}:r>m$. If the null hypothesis is rejected, the next stage is performed, and if the null hypothesis is accepted, the number of confirmed cointegrating relationships is *m*.

Next, the found cointegrating vectors are normalized, such that:

(15) $$\beta ={\left[-1,-\beta_{2},\ldots ,-\beta_{n}\right]}^{T}$$

and the residuals of projecting the data series onto the cointegrating vectors is tested for the existence of the unit root. If that test confirms that the residuals are stationary, the cointegration relationship is confirmed.

### 2.3. The Specimens

**Figure 3:**
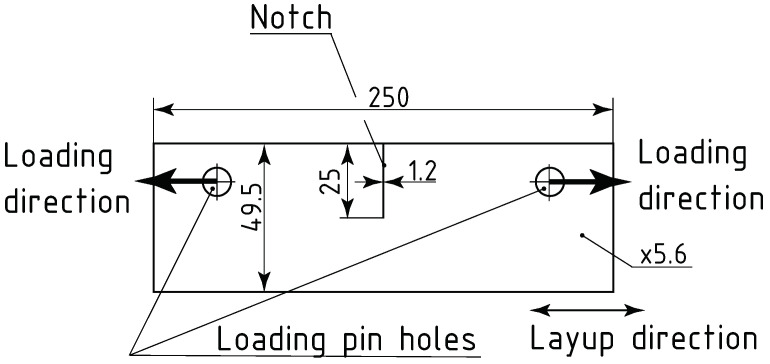
Specimen dimensions and tensile test setup schematics.

**Figure 4:**
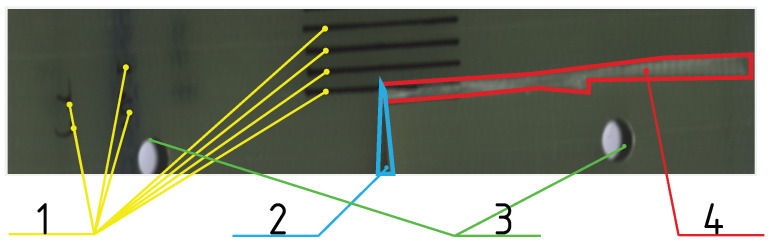
Photograph of the specimen after the introduction of the crack, with different regions of the specimen highlighted: 1—Markings inked on the surface of the specimen, for purpose of specimen identification, and as a size reference. 2—Holes cut into the specimen before the tensile test, for purpose of accepting loading pins. 3—Notch cut into the specimen before the tensile test, directed perpendicularly to the glass fibre layup. 4—Area damaged in tensile test—a crack going through the whole thickness of the specimen, starting at the bottom of the notch, and ending at the edge of the specimen, following the layup direction of glass fibres.

The specimens used for this study were made of a glass fibre-reinforced plastic (GFRP) composite material. The matrix consisted of an epoxy resin, the glass fibres had a unidirectional layup, and there were five layers of the glass fibre mat. The material data for such composites, for the purpose of simulating the damage event, can be found in the literature [33,34]. To introduce the damage, a destructive strength test was performed. The specimen was subjected to a quasi-static tensile test, in accordance with the ASTM E1922 Standard [35]. Test Method for Translaminar Fracture Toughness of Laminated and Pultruded Polymer Matrix Composite Materials. The test consisted of loading the specimen in tension, along the direction of the fibre layup, as presented on Figure 3. The load was applied by the loading pins that were put into the loading holes of the specimen. The test was displacement driven, and the loading rate was set at 3 mm min ( $3\times 10^{-5}$ m s). The test was stopped when the material failure was confirmed by a rapid drop of force and visual confirmation of the presence of a crack. The peak force registered was 9639.6 N. As a result of the test, a crack of length 120 mm and width of 4 mm, and reaching the full depth of the specimen, directed along the fibre layup direction, was introduced to the specimen, as shown in Figure 4.

### 2.4. Hyperspectral Imaging Equipment

After the damage was introduced, a hyperspectral image of the specimen was captured. Imaging was completed using a Headwall Photonics Inc., Bolton, USA Desktop Scanning Kit. The hyperspectral camera was equipped with two imaging sensors: a visible and near infrared (VNiR) H-type sensor, and a short-wave infrared (SWiR) M-type sensor. The VNiR sensor has a wavelength range of 400 nm to 1000 nm with a spectral resolution of 1.6 nm, and spatial resolution of 1600 px, and the SWiR sensor has a wavelength range of 890 nm to 2500 nm, with a spectral resolution of 9.8 nm and spatial resolution of 384 px.

The Desktop Scanning Kit utilizes a pushbroom scanning method, i.e., a single row of an image is registered at regular intervals, while the scanned object is moved at a setup rate by the motion platform integrated into the device.

To improve the accuracy of the upcoming image registration step, fiducial markers were placed in the background of the scanned specimen.

As a result, two hyperspectral data cubes were obtained, one from each sensor, along with white and black references for image calibration purposes. The VNiR hypercube dimensions were 1600×2500×375, and the SWiR hypercube dimensions were 384×600×165.

### 2.5. Hyperspectral Image Preprocessing

The first step of preprocessing consists of converting the digital number (DN) registered by the sensors to a physical quantity of reflectance. This step is called the calibration of a hyperspectral image [36]. Reflectance (*R*) is a dimensionless measure of an effectiveness in reflecting the radiant energy. It is defined as:

(16) $$R=\frac{\Phi_{e}^{r}}{\Phi_{e}^{i}}$$

where $\Phi_{e}^{r}$ is the radiant flux reflected by the surface, and $\Phi_{e}^{i}$ is the radiant flux incident onto that surface.

Black and white references obtained during the imaging are themselves hyperspectral images of size 1×1×λ, and are used in the calibration procedure to obtain reflectance values of pixels in a hyperspectral image that are absolute—independent of the radiant flux of an illuminator used during the imaging step, or the exposition time of an image. The white reference is obtained by capturing a hyperspectral image of a calibration standard made of Spectralon material, which is highly reflective at every wavelength used by hyperspectral sensors. The black reference is obtained by capturing a hyperspectral image with the sensors covered by a shield impermeable to electromagnetic radiation at wavelengths used by hyperspectral sensors.

With those two references, an image is calibrated by applying:

(17) $$R\left(x,y,\lambda \right)=\frac{DN\left(x,y,\lambda \right)-DN_{black}\left(\lambda \right)}{DN_{white}\left(\lambda \right)-DN_{black}\left(\lambda \right)}$$

where R(x,y,λ) is a reflectance of a hyperspectral image pixel at *x* and *y* coordinates at λ wavelength, DN(x,y,λ) is a digital number of a hyperspectral image pixel at *x* and *y* coordinates at λ wavelength, $DN_{black}\left(\lambda \right)$ is a digital number of the black reference at λ wavelength, and $DN_{white}\left(\lambda \right)$ is a digital number of the white reference at λ wavelength. The calibration is performed on both VNiR and SWiR hypercubes independently.

The next step in the preprocessing procedure is image registration [37]. In this step, both hypercubes are prepared for fusion by aligning the image and upscaling the SWiR hypercube to match the spatial resolution of the VNiR hypercube. Three corresponding pairs of points are chosen from both hypercubes by selecting the corners of fiducial markers. Their coordinates are stored in vectors $x_{v}$ and $y_{v}$ for the VNiR hypercube and $x_{s}$ and $y_{s}$ for the SWiR hypercube. Using these two vectors, an affine transformation is calculated, such that

(18) $$x_{vi}y_{vi}=Ax_{si}y_{si}+B\;\forall \;i$$

where A is a transformation matrix, B is a translation vector, and *i* is the number of pixels. That transformation is then applied to the SWiR hypercube.

After the registration and fusion, a splice correction is performed [38], which shifts the reflectance values in the SWiR hypercube to satisfy:

(19) $$R_{VNiR}\left(x,y,\lambda_{n}\right)=R_{SWiR}\left(x,y,\lambda_{n}\right)$$

where $\lambda_{n}$ is the wavelength in the middle of the wavelength range covered by both the VNiR and SWiR sensors, in our case $\lambda_{n}=$ 945 nm. The correction is performed by applying:

(20) $$R_{SWiR\_corrected}\left(x,y,\lambda \right)=R_{SWiR}\left(x,y,\lambda \right)-f\left(\lambda \right)$$

where f(λ) is a correction factor given by:

(21) $$f\left(\lambda \right)=R_{SWiR}\left(x,y,\lambda_{n}\right)-\left(2\;\cdot \;R_{VNiR}\left(x,y,\lambda_{n}\right)-R_{VNiR}\left(x,y,\lambda_{n-1}\right)\right)$$

After applying the splice correction, the VNiR and SWiR hypercubes are fused, which is obtained by concatenation along the spectral dimension.

The next step of preprocessing is an application of the Savitzky–Golay filter for each pixel along the spectral dimension [39]. The window size used was 7, and the order of the filter was 2. The filtration was achieved by applying:

(22) $$\begin{array}{c} R_{filtered}\left(x,y,\lambda \right)=(-2R\left(x,y,\lambda -3\right)+3R\left(x,y,\lambda -2\right)+6R\left(x,y,\lambda -1\right)+\\ +7R(x,y,\lambda )+6R(x,y,\lambda +1)+3R(x,y,\lambda +2)-2R(x,y,\lambda +3))/21 \end{array}$$

After the filtration, 10 points were selected from the damaged region and 10 from the undamaged region of the specimen. By calculating the means of these points, spectral signatures of the damage to the composite and of the healthy material were constructed.

### 2.6. Data Conditioning Using Cointegration Analysis

The cointegration analysis is used in the data conditioning step as a trend removal tool [40]. The Johansen procedure was applied to find the cointegrating vector β for the spectral signatures of healthy and damaged material. The lag order used in the AR models of signatures in the Johansen test was N=7. The t-statistic of −3.46 exceeded a critical value of the Dickey–Fuller t-distribution at a confidence level of 95%: −3.42 allowed us to conclude that a cointegration relationship between these spectral signatures exists.

The whole hyperspectral image was then projected onto the found cointegrating vector β by applying:

(23) $$R_{coint}\left(x,y,\lambda \right)=\beta \left(\lambda \right)\times R\left(x,y,\lambda \right)$$

The resulting data cube was then subtracted from the fused and filtered hypercube, resulting in a datacube of cointegration residuals.

(24) $$R_{residuals}\left(x,y,\lambda \right)=R\left(x,y,\lambda \right)-R_{coint}\left(x,y,\lambda \right)$$

To allow for the comparison of the results of applying the data conditioning technique, two independent workflows for processing hyperspectral images for the purpose of damage detection will be examined: First, the method based on traditional hyperspectral image processing, where the learning data are simply the spectra of the pixels on the hyperspectral image, and second, a method where cointegration analysis is used to transform the spectra into residuals of cointegration. For this purpose, the trend removal and filtering subroutines in the traditional workflow are skipped to allow the ADF test, which is required to be positive and indicate the presence of the unit root in raw data, to hint as to the existence of cointegrating vectors. After this step, the hypercube is projected onto these vectors, which allows for the calculation of cointegration residuals. These residuals are used as the learning data in our proposed method.

### 2.7. Classification Algorithms

Three kinds of classification algorithms were used, and with every one of them, the learning and classification was performed once on a filtered hypercube, and once on a data cube of cointegration residuals. The first algorithm was a multilayer perceptron neural network. It consisted of 10 input neurons, 2 fully connected layers of 10 neurons, and two output neurons. The second algorithm was a support vector machine with Gaussian kernel. The last algorithm was a support vector machine with a cubic kernel. Before the classification, a principal component analysis was performed, and the first 10 principal components were used as the training data.

## 3. Results

### 3.1. Data Conditioning Results

The ADF tests were performed on the spectral signatures obtained from the preprocessing of hyperspectral images. The null hypothesis in ADF was $H_{0}:\;\delta_{0}=0$, and the alternative was $H_{1}:\;\delta_{0}<0$. The results of the ADF tests, along with the critical values of DF distribution, are presented in Table 1.

The models used in the ADF tests were AR(5) with drift. In both cases, $H_{0}$ was not rejected, confirming that both spectral signatures are integrated of order I(1).

After the ADF test, the Johansen test for cointegration was performed. Similarly, a VECM model with lag order of 5 was used. The null hypothesis in the Johansen test was $H_{0}:\;r<k$, where r is the number of cointegrating vectors, and k is the number of tested data series. The alternative hypothesis was $H_{1}:\;r=k$. The results of the tests are presented in Table 2.

For VECM models with either a constant, or a trend, the tests indicate that there is one cointegrating vector. The models with no deterministic terms, and one with both a constant and a trend, indicate that there are two valid cointegrating vectors.

**Figure 5:**
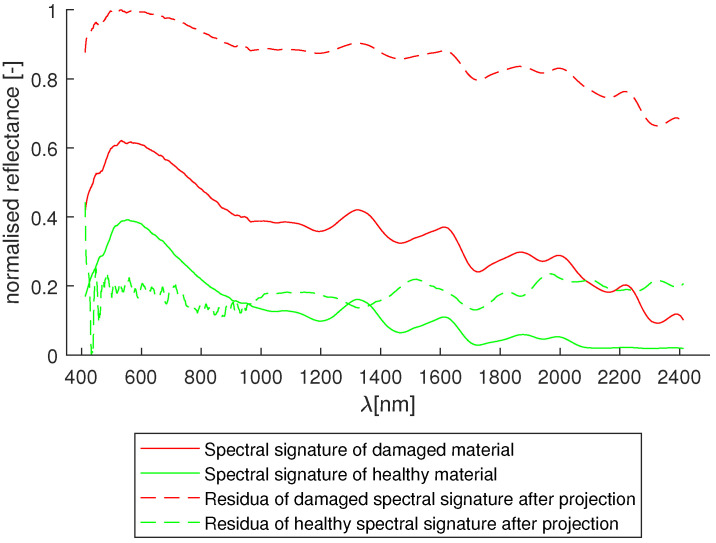
Model spectra before and after the data conditioning step.

The cointegrating vector was calculated using the model with the deterministic trend. The original spectral signatures, and the residua of projecting them onto the cointegrating vectors, are shown in Figure 5.

### 3.2. Classification without the Data Conditioning

**Figure 6:**
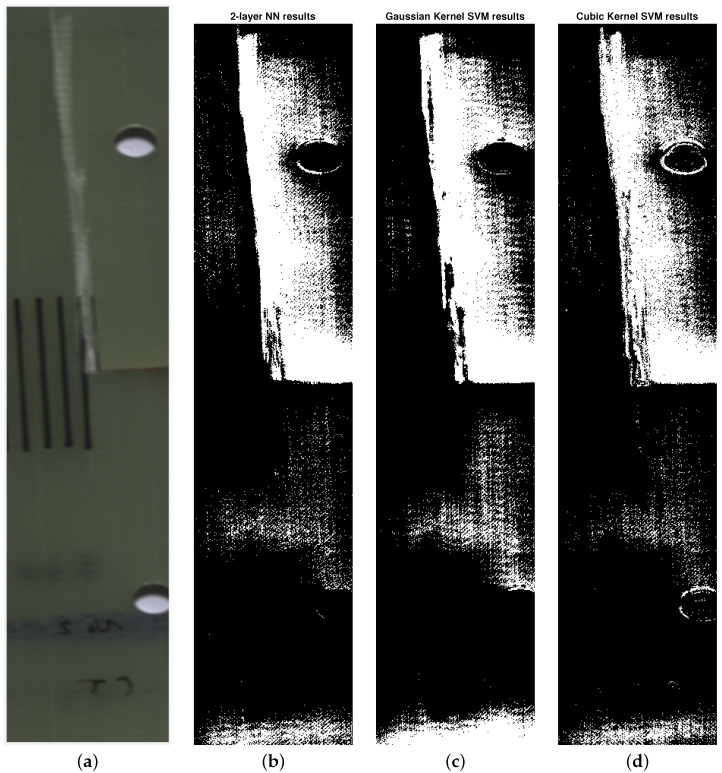
Classification results without the cointegration analysis data conditioning. (**a**): A reference RGB image of the damaged specimen. (**b**): Results of classification using 2-layered neural network. (**c**): Results of classification using SVM with Gaussian kernel. (**d**): Results of classification using SVM with cubic kernel.

The classification was performed using three kinds of algorithms. The learning and learning validation data consisted of 500 spectral signatures of healthy and damaged material, which defined the two classes. A five-fold cross-validation was used to prevent overfitting. After the learning was completed, the classification algorithms were applied to the preprocessed hyperspectral images. The results of classification are presented in Figure 6, and the metrics of classifier performance in Table 3.

The accuracies achieved by the classification algorithms in the validation step were 81.4% for the bilayered artificial neural network, 76.4% for the SVM with Gaussian kernel, and 83.6% for the SVM with cubic kernel.

### 3.3. Classification with the Data Conditioning

**Figure 7:**
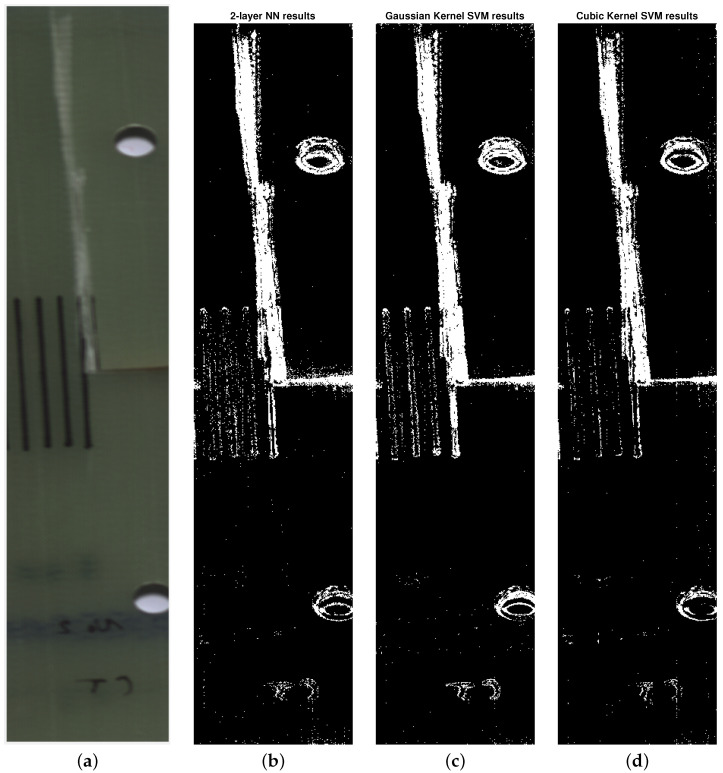
Classification results with the cointegration analysis data conditioning. (**a**): A reference RGB image of the damaged specimen. (**b**): Results of classification using 2-layered neural network. (**c**): Results of classification using SVM with Gaussian kernel. (**d**): Results of classification using SVM with cubic kernel.

The setup for the classification algorithms was similar to that described in a previous section. The key difference is that instead of using spectral signatures as the algorithms’ input, the residuals of the projection onto the cointegrating vectors were used. The results of classification are presented in Figure 7, and the metrics of the classifiers are shown in Table 4.

The accuracies achieved by the classification algorithms in the validation step were 94.5% for the bilayered artificial neural network, 94.2% for the SVM with Gaussian kernel, and 94.5% for the SVM with cubic kernel.

## 4. Discussion

The ADF tests confirmed that both of the spectral signatures are non-stationary, and are integrated of order I(1). Because the variant of the ADF test used utilized the AR(5) model with drift, both of these signals can be classified as trend-stationary. Because of the cointegration analysis’ proven ability to detrend data series, it is reasonable to apply the Johansen test in an effort to remove those trends.

The noteworthy difference in such application is that the cointegration and the concept of stationarity itself in our research is applied not to a time series data, but to the spectra of particular areas on the hyperspectral image, or the models of their spectral signatures—in other words, the temporal dimension is replaced with a spectral dimension.

While it is not always obvious which features, or their combinations, of the data used in the machine learning are the key factors in the determination of the algorithm’s output, the increase in the distance between the different classes generally improve the performance. This is supported by the remarkable success of the PCA analysis in nearly every machine learning task, and other methods that achieve a similar result, for example, application of the kernel tricks in SVM algorithms.

The proposed usage of cointegration analysis achieves that goal, which is visible in the difference between the input data in Figure 5. Another confirmation of the proposed algorithm’s validity comes from the classification results themselves. By comparing the results of classification with and without the data conditioning step in Figure 6 and Figure 7, a conclusion can be drawn that the algorithms that used cointegration residuals perform better than the ones that use the spectral signatures. In the first case, the areas classified as damaged are consistent with the reference image, and the false positives that are present appear as the small groups of pixels, and can be distinguished easily from the actual damage. In the latter case, the damaged areas have been correctly classified as damaged; however, the bounds of the damaged area are hard to determine exactly because of the difference in its characteristic—on the left side of the crack, the transition from healthy to damaged is clear and abrupt, but on its right side, the transition seems gradual and soft. This difference can be attributed to the existence of trends in the data, which are abundant in hyperspectral imaging due to imperfect lighting conditions.

Overall, the improvement in performance of the classifiers when using the data conditioning step is most visible in the increase in the precision metric. Across all the tested classifiers, the precision increased twofold, which is the result of significantly fewer occurrences of false positive classifications. The consistency of this increase in each of the three tested classification methods: the artificial neural network, and the Gaussian and cubic kernel SVMs, suggests that the improved quality of classification by using the proposed method of data conditioning is not specific to a singular classifier and is beneficial regardless of the type of classifier used.

## 5. Conclusions

In our study, the cointegration analysis was used to develop an unconventional data conditioning process for classification of hyperspectral images. The process takes advantage of cointegration analysis’ ability to remove trends in the data, which were suspected to decrease the performance of classifiers. Application of the developed technique, and comparison of the results, obtained with and without its use, allowed us to conclude the following:

- The application of cointegration analysis improves the results of classification, especially in regard to decreasing the number and spatial distribution of false positives;
- In hyperspectral imaging, the benefits of data conditioning apply universally to classification algorithms, regardless of the specific algorithm used;
- In hyperspectral imaging, the concept of signal stationarity can be considered in the spectral domain, instead of the traditional temporal domain, while still keeping its properties.

This said, we believe that further work is required on the topic of the use of cointegration analysis in HSI. Among many other areas, the aspects of the performance against different data conditioning procedures, other approaches to damage detection using HSI (such as using segmentation algorithms for deciding if the pixel in question bears marks of damage or not), the performance on different kinds of samples (different kinds of damage, different kinds of material), and the applicability to the detection of dynamic crack propagation can be explored further.

## Figures and Tables

**Table 1:** Results of ADF test at significance level of 5%.

Data Series	T-Statistic	Critical Value	Null Rejected
Damaged spectral signature	−0.5655	−2.8681	False
Healthy spectral signature	−2.2881	−2.8681	False

**Table 2:** Johansen test results by type of VECM model, at significance level of 5%, at r = 2.

VECM Model	T-Statistic	Critical Value	Null Rejected
No deterministic terms	17.9372	12.3206	True
With deterministic constant	18.2173	20.2619	False
With deterministic trend	14.1937	15.4948	False
With deterministic trend and constant	37.8892	25.8723	True

**Table 3:** Classifier metrics without data conditioning.

	Sensitivity	Specificity	Precision	Accuracy	F1 Score
Neural network	0.695	0.824	0.263	0.814	0.381
Gaussian kernel SVM	0.618	0.777	0.199	0.764	0.301
Cubic kernel SVM	0.713	0.847	0.296	0.836	0.418

**Table 4:** Classifier metrics with data conditioning, and the trend in comparison to metrics without data conditioning.

	Sensitivity	Specificity	Precision	Accuracy	F1 Score
Neural network	0.670↘	0.970↗	0.672↗	0.945↗	0.674↗
Gaussian kernel SVM	0.695↗	0.968↗	0.649↗	0.942↗	0.654↗
Cubic kernel SVM	0.587↘	0.977↗	0.698↗	0.945↗	0.638↗

## Data Availability

Dataset available on request from the authors.

## Abbreviations

The following abbreviations are used in this manuscript:

ACE	Adaptive Cosine Estimator
ADF	Augmented Dickey–Fuller test
ANN	Artificial neural network
AR	Autoregressive
DF	Dickey–Fuller test
DN	Digital Number
GFRP	Glass Fibre-Reinforced Plastic
HSI	Hyperspectral Imaging
NDT	Non-destructive Testing
NiR	Near Infrared
PDF	Probability Density Function
SHM	Structural Health Monitoring
SVM	Support vector machine
SWiR	Short-Wave Infrared
VECM	Vector Error Correction Model
VNiR	Visible and Near Infrared
